# 0592. Metabolic acidosis induced by haemorrhage and hydrochloric acid generates different cardiorespiratory responses

**DOI:** 10.1186/2197-425X-2-S1-P36

**Published:** 2014-09-26

**Authors:** G Sabbatini, A Dyson, M Singer

**Affiliations:** University College London, Bloomsbury Institute of Intensive Care Medicine, London, United Kingdom

## Introduction

Metabolic acidosis is classically thought to induce an enhanced ventilatory pattern, irrespective of the underlying aetiology.

## Objectives

To induce a similar level of acidaemia in a rat model, by either infusion of an acidic solution or by blood withdrawal, and to assess the physiological responses to these insults.

## Methods

Isoflurane-anaesthetised, tracheotomized rats were instrumented with left common carotid arterial and right jugular venous lines for blood sampling/BP monitoring and fluid/blood administration, respectively. Oxylite^TM^ probes (Oxford Optronix, UK) placed in thigh muscle were used to monitor tissue oxygen tension (tPO_2_). Animals were subjected to either continuous 0.1 M hydrochloric acid (HCl) infusion or 60% withdrawal of estimated blood volume in six 10% steps over three hours to induce an equivalent fall in arterial base excess (BE). All animals (including a control group) received n-saline throughout. Hourly measurements were made of haemodynamics, tPO_2_ and arterial blood gas analysis.

## Results

See figure 1.

**Figure 1:**
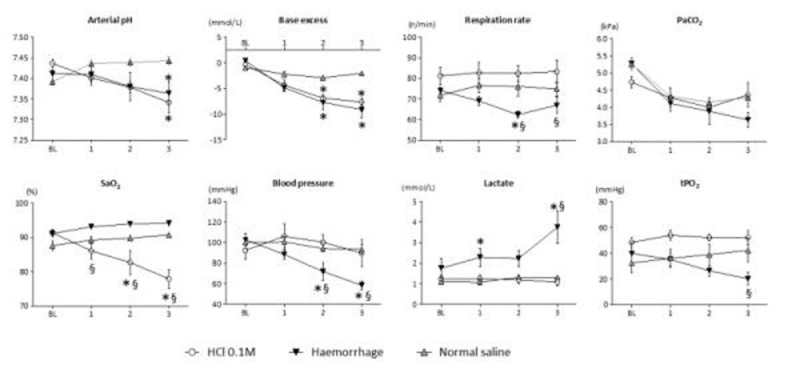
Results. BL= baseline, SaO_2_= arterial oxygen saturation. Data shown as mean (SEM). Hydrochloric acid (HCl) n=6, Haemorrhage n=8, Controls n=10/group, *p< 0.05 comparing treated to controls, §p< 0.05 comparing haemorrhage to HCl. Statistics: repeated measures two-way ANOVA and Bonferroni's test for multiple comparisons.

HCl induced a metabolic acidosis with arterial hypoxaemia yet a preserved muscle tPO_2_, no tachypnoea nor fall in PaCO_2_. By contrast, haemorrhage to achieve a similar acidaemia, resulted in significant falls in blood pressure and tPO_2_, hyperlactataemia, a small rise in SaO_2_ and a decrease in respiration rate with a concomitant fall in PaCO_2_ probably related to higher tidal volumes.

## Conclusions

Tissue hypoperfusion (and not just acidaemia per se) is an important component that triggers an enhanced ventilatory drive.

